# The role of vitamin D in subjective tinnitus—A case-control study

**DOI:** 10.1371/journal.pone.0255482

**Published:** 2021-08-18

**Authors:** Magdalena Nowaczewska, Stanisław Osiński, Maria Marzec, Michał Wiciński, Katarzyna Bilicka, Wojciech Kaźmierczak

**Affiliations:** 1 Department of Otolaryngology, Head and Neck Surgery, and Laryngological Oncology, Ludwik, Rydygier Collegium Medicum in Bydgoszcz, Nicolaus Copernicus University, Bydgoszcz, Poland; 2 Department of Pharmacology and Therapeutics, Faculty of Medicine, Collegium Medicum in Bydgoszcz, Nicolaus Copernicus University, Bydgoszcz, Poland; 3 Department of Sensory Organs Examination, Faculty of Health Sciences, Collegium Medicum in Bydgoszcz, Nicolaus Copernicus University, Bydgoszcz, Poland; Universidade Federal de Sao Paulo/Escola Paulista de Medicina (Unifesp/epm), BRAZIL

## Abstract

Regarding the high prevalence of vitamin D (25(OH)D) deficiency in the population and its possible association with ear diseases, we aimed to investigate the 25(OH)Dserum level in patients with subjective, nonpulsating tinnitus and its effect on tinnitus severity. The study included 201 tinnitus patients and 99 controls. Patient clinical information, including tinnitus characteristics and severity according to Tinnitus Handicap Inventory (THI), loudness assessed by Visual Analogue Scale (VAS), audiometry, and the blood level of vitamin D, was recorded. The level of 25(OH)D in tinnitus patients was significantly decreased compared with the controls (19.86 ± 7.53 and 27.43 ± 8.85 ng/ml, respectively; P value < 0.0001). More patients in the tinnitus group were deficient in vitamin D, compared with the controls (50.7% vs. 22.2% respectively, p < 0.0001). Tinnitus patients with a lower serum level of 25(OH)D (≤15 ng/dl) were significantly younger, had a higher degree of tinnitus severity measured with THI and VAS scales, had higher triglyceride and TSH levels, and a lower HDL level compared with individuals who had higher 25(OH)D level (>15 ng/dl). There was a strong correlation between the 25(OH)D level and THI. Our findings suggest that a large proportion of tinnitus patients suffers from vitamin D deficiency and that the vitamin D level correlates with tinnitus impact. We recommend a vitamin D assessment for all tinnitus patients.

## 1. Introduction

Tinnitus is a very prevalent condition, defined as a perception of sound or noise in the absence of an external source. It affects millions of people worldwide, often coexists with mood disorders, and impairs cognitive function, thus significantly diminishing the quality of life and placing a considerable burden on society, primarily due to financial repercussions of treatment cost [1, 2]. The prevalence of tinnitus in the general population ranges between 10% and 15% and increases with age [3]. Tinnitus can occur in association with several disorders, including otologic diseases, acoustic trauma, metabolic and neurological diseases, or stress; however, most of the cases remain idiopathic [4, 5]. Regardless of many efforts and research, the pathophysiology of this disease remains poorly understood. Many studies indicate that subjective tinnitus starts in the central auditory structures due to neuroplastic adaptations that occur in response to changes in the peripheral auditory system [6, 7]. Neural changes arise at the level of synapses between the auditory nerve and inner hair cells and within multiple levels of the central auditory pathway. The long-term maintenance of tinnitus is probably a function of a complex network of structures involving central auditory and non-auditory systems [8]. In most cases, tinnitus is believed to be associated with some degree of cochlear damage [9]. Due to the unclear pathogenesis, the current tinnitus treatments are diversified, and up to now, there is no effective medication for tinnitus [10]. Regardless of limited data, the use of supplements in tinnitus treatment is very common [11, 12]. Vitamin D deficiency is an emerging global health problem, affecting approximately 30%–80% of children and adults worldwide [13]. It is worth noting that vitamin D has numerous functions in the body, far beyond its classical effects on skeletal mineral homeostasis. Apart from its important role in calcium homeostasis and metabolism, vitamin D also diminishes inflammation, modulates cell growth, and controls the neuromuscular and immune systems [13, 14]. Moreover, vitamin D deficiency has been linked to many diseases including infections, autoimmune and cardiovascular diseases, neuromuscular, musculoskeletal, and psychiatric disorders, diabetes, cancers, pain, and headaches [13, 15]. Regarding the presence of vitamin D receptors in the inner ear, one should expect that vitamin D deficiency may influence vestibular and auditory function [16, 17]. In fact, recent studies have reported the high prevalence of vitamin D deficiency in patients with inner ear diseases, including benign paroxysmal positional vertigo, Menière’s disease, vestibular neuritis, idiopathic facial paralysis, and idiopathic acute hearing loss [18–20]. The role of vitamin D in the inner ear diseases may be related to calcium metabolism, fluids, and nerve transmission impairment leading to the degeneration of auditory structures, demineralization of the cochlea, cochlear sensitivity to chronic ischemic effects, and lysosomal enzymes imbalance [16, 17, 20].

Despite the clear link between vitamin D and ear diseases, there is a lack of data regarding the influence of vitamin D on tinnitus. Thus, in the present study, we aimed to further examine the relationship between vitamin D and tinnitus, to determine the prevalence of vitamin D deficiency in patients with tinnitus and its effect on tinnitus parameters, especially its severity.

## 2. Materials and methods

### 2.1 Patients

This prospective study involved 201 patients recruited consecutively from the Department of Otolaryngology in University Hospital in Bydgoszcz, from February 2019 to February 2020. Patients were diagnosed with subjective, nonpulsating tinnitus. The control group included 99 volunteers matched according to age and sex, without tinnitus and other ear diseases, with no hearing loss (every frequency in pure tone audiometry ≤25 dBHL), who were not on vitamin D supplementation. All patients and controls were white skin and caucasian race. We recruited controls by local advertisement. We recruited the patients and controls proportionally during one year (stable number of patients and controls each month). During hospitalization, all tinnitus patients were thoroughly assessed by the multidisciplinary team consisting of otorhinolaryngologists, neurologists, and audiologists. Complete anamnesis as well as otological and neurological examination were applied to all tinnitus patients. They were asked about tinnitus onset and related clinical factors, the presence of comorbidities, and additional medical history. Other information about tinnitus was collected, including perceptional characteristics of the tinnitus sound, temporal properties (continuous, intermittent), location (in one or both ears, or in the head), and severity. Patients were asked about the presence of vertigo and headache (the type of headache was diagnosed according to the third edition of the International Classification of Headache Disorders (ICHD-3) [21]. Data about coexisting hypertension, diabetes, smoking, mood, sleep, and thyroid disorders were also collected. A routine blood test included the vitamin D serum level, thyroid function tests, testosterone and estrogen levels, and lipid profiles. Based on recommendations for Central Europe, the ranges of serum 25 D concentration were defined as deficiency (<20 ng/mL), insufficiency (20–30 ng/mL), and optimal concentration (>30 ng/mL) [22]. All tinnitus patients underwent Carotid Doppler ultrasonography with intima-media complex (IM complex) assessment.

#### 2.1.1 Exclusion criteria for tinnitus group

Pulsating tinnitus, obvious local and systemic acute inflammation, tumor history, ear-surgery-related diseases, serious medical illness, chronic conditions (asthma or allergies, inflammatory diseases of connective tissue, gastrointestinal disorders, diseases of kidneys and liver, disorders of bone metabolism); administration of any preparation containing vitamin D, during six months preceding the study, use of medication that influence vitamin D status.

#### 2.1.2 Exclusion criteria for control group

Present tinnitus or history of tinnitus, ear disease, hearing loss, obvious local and systemic acute inflammation, tumor history, ear-surgery-related diseases, serious medical illness, chronic conditions (asthma or allergies, inflammatory diseases of connective tissue, gastrointestinal disorders, diseases of kidneys and liver, disorders of bone metabolism); administration of any preparation containing vitamin D, during six months preceding the study, use of medication that influence vitamin D status.

All the procedures were approved by the Local Ethics Committee of the Ludwik Rydygier Collegium Medicum in Bydgoszcz (approval number KB 219/2019). The subjects gave their informed, written consent before the start of any procedure.

### 2.2 Tinnitus

The degree of perceived tinnitus severity was measured according to the validated Polish version of the Tinnitus Handicap Inventory (THI), while tinnitus loudness was assessed by the Visual Analogue Scale (VAS) for tinnitus loudness [23]. VAS scores were performed by asking the patient to rate the loudness of tinnitus from 0 to 10. Psychoacoustic characteristics of tinnitus including its loudness and pitch were measured using the standard clinical method by presenting sounds similar to those described by the patient [24, 25]. All patients underwent a familiarization procedure before the test. Tinnitus matching was performed using audiometry. Test frequencies were performed, included frequencies ranging from 250 to 16,000 Hz. After hearing sounds with different loudness and pitch, the patients indicated that one more closely resembled their tinnitus. In cases of unilateral tinnitus, patients received the test sound to the contralateral ear, while those with bilateral tinnitus had the sound offered to the ear with lower tinnitus loudness. If the tinnitus was symmetrical or experienced in the head, the patient themselves selected the ear to be tested. First, the 1000 Hz pure tone of 10 dB sounds was produced on the ear and the frequency was changed until the patients considered the sound to be closest to his/her tinnitus. Second, the loudness of the sound was adjusted until the sound was similar to the loudness of his/her tinnitus. Three measures of frequency and loudness each were performed, and the average of three repeated measurements was used in this process.

### 2.3 Audiometry

Patients were evaluated using pure tone audiometry in an acoustically treated booth to test frequencies up to 16 kHz (audiometer, Interacoustics). Sensorineural hearing loss was defined according to WHO criteria, in the better ear, as an average of 500, 1000, 2000, and 4000 Hz., and was graded as slight (26–40 dBHL), moderate (41–60 dBHL), severe (61–80 dBHL), or profound (81 dBHL or greater) [26]. If a patient had unilateral hearing loss, it was also graded as above. High frequency hearing loss was defined as an average of 2000, 4000, and 8000 Hz above 25 dB.

### 2.4 Statistics

Statistical analysis was performed using Microsoft Excel and RStudio software. Statistical significance was considered when p < 0.05. The Shapiro-Wilk test was used to assess the normality of each parameter’s distribution. For parameters on the nominal level, to evaluate p-value chi square test was performed. For other parameters, differences between the means were calculated using a one-way ANOVA (analysis of variance) test (in the case of analyzing three groups) or a Student’s T-test (in the case of analyzing two groups). When the analyzed variable was not normally distributed, a Wilcoxon signed rank test was performed. For regression model, the process of selecting the optimal set of prognostic factors was performed using a backward selection procedure, starting with the model with all potential prognostic factors and eliminating irrelevant variables in subsequent steps. No sample size has been determined at the start of the study.

## 3. Results and discussion

In total, 201 patients with tinnitus and 99 controls were enrolled in this study, over a period of one year. The two groups did not present statistically significant differences in age and sex distribution. The tinnitus group consisted of 93 males and 108 females, with a mean age of 49.9 years (a range of 19–76 years). Mean tinnitus duration was 4.7 years. Tinnitus was unilateral in 52.2% of patients, bilateral in 47.8%, and was heard in the head in 17.4% of patients. Constant tinnitus was experienced by 47.8% of patients, while in 52.2% tinnitus was intermittent. In 56 patients (32.5%), tinnitus was associated with objective hearing loss. In 56.7% of patients, it was associated with vertigo, and in 46.3% of patients, it was associated with headache. The average hearing level of tinnitus matched at their tinnitus pitch was 42.27 dB. THI scores were 41.14 on average and the mean tinnitus loudness measured by the VAS scale was 6.38. The characteristics of the patients and controls are presented in Table 1., while the clinical characteristics of the whole tinnitus group, and depending on 25(OH)D blood level are placed in Table 2.

**Table 1:** Demographic characteristics and 25(OH)D blood level of the tinnitus group and controls.

Parameters	Tinnitus Group n = 201	Controls n = 99	p-Value
Age (mean ± SD)	49.9±13.2	48.3±17.5	0.411
BMI	24,5	25,1	0.743
Gender (male/female)	93/108	48/51	0.811
Vitamin D level (ng/dl)	19.86 ± 7.53	27.43 ± 8.85	**<0.0001**
Vitamin D level range			
Optimal, n (%)	17 (8.5%)	37 (37.2%)	**<0.0001**
Insufficiency, n (%)	82 (40.8%)	40 (40.4%)	1
Deficiency, n (%)	102 (50.7%)	22 (22.2%)	**<0.0001**

**Table 2:** Demographic and clinical characteristics of the whole tinnitus group, and depending on 25(OH)D level.

	Whole group information	Comparison between 25(OH)D groups		
Parameters	Tinnitus group n = 201	Tinnitus group 25(OH)D level ≤ 15 N = 59	Tinnitus group 25(OH)D level >15 N = 142	p-value
Age (mean ± SD)	49.9±13.2	46.68 ±12.64	51.23 ±13.23	**0.024**
Duration of tinnitus (years)	4.7±5.5	4.47 ±5.27	4.8 ±5.56	0.597
Tinnitus localization				
bilateral, n (%)	96 (47.8%)	31 (52.5%)	65 (45.8%)	0.439
unilateral, n (%)	105 (52.2%)	28 (47.5%)	77 (54.2%)	0.308
ear, n (%)	166 (82.6%)	46 (78.0%)	120 (84.5%)	
head, n (%)	35 (17.4%)	13 (22.0%)	22 (15.5%)	
Tinnitus continuous, n (%)	161 (80.1%)	44 (74.6%)	117 (82.4%)	0.245
Tinnitus intermittent, n (%)	40 (19.9%)	15 (25.4%)	25 (17.6%)	
VAS mean	6.38±2.40	6.90 ±2.45	6.16 ±2.36	**0.038**
THI mean	41.14±27.31	59.73 ±26.66	33.42 ±23.68	**<0.001**
Loudness (dB)	42.27±22.53	42.91 ±22.44	42.02 ±22.65	0.999
Frequency (Hz)	3137±277	3171.36 ±2791.64	3123.37 ±2776.34	0.741
Hearing loss, n (%)	34 (16.9%)	11 (18.6%)	23 (16.2%)	0.681
Hearing loss grade				0.681
Slight, n (%)	21 (61.8%)	8 (72.7%)	13 (56.5%)	
Moderate, n (%)	10 (29.4%)	3 (27.3%)	7 (30.4%)	
Severe, n (%)	2 (5.9%)	0 (0%)	2(8.7%)	
Profound, n (%)	1 (2.9%)	0 (0%)	1 (4.3%)	
High frequency hearing loss, n (%)	103 (51.2%)	28 (47.5%)	75 (52.8%)	0.6431
Vertigo, n (%)	114 (56.7%)	33 (55.9%)	81 (57.0%)	1
Headache, n (%)	93 (46.3%)	27 (45.8%)	66 (46.5%)	1
Migraine, n (%)	28 (30.1%)	7 (26.9%)	6 (7.8%)	
TTH, n (%)	52 (55.9%)	8 (30.8%)	20 (26.3%)	
Other, n (%)	13 (14.0%)	12 (46.2%)	40 (52.6%)	
Sleep disorder, n (%)	47 (23.4%)	12 (20.3%)	35 (24.6%)	0.586
Depression, n (%)	53 (26.4%)	15 (25.4%)	38 (26.8%)	1
Anxiety, n (%)	24 (11.9%)	6 (10.2%)	18 (12.7%)	0.812
Hypertension, n (%)	62 (30.8%)	17 (28.8%)	45 (31.7%)	0.740
Smoking, n (%)	23 (11.4%)	5 (8.5%)	18 (12.7%)	0.473
Diabetes, n (%)	18 (9.0%)	8 (13.6%)	9 (6.3%)	0.102
Thyroid disease, n (%)	24 (11.9%)	8 (13.6%)	16 (11.3%),	0.639
Cholesterol, mg/dL	187.43±36.65	188.27 ±39.34	187.07 ±35.62	0.846
Triglyceride, mg/dL	124.03±68.06	145.83 ±91.46	114.96 ±53.38	**0.042**
HDL, mg/dL	52.28±14.57	49.69 ±15.61	53.35 ±14.03	0.068
LDL, mg/dL	120.40±34.24	122.76 ±33.79	119.35 ±34.49	0.516
Testosterone level, ng/dL				
Women	31.12±11.28	29.38 ±10.56	31.89 ±11.57	0.530
Men	502.0±344.4	533.15 ±604.81	489.74 ±157.57	0.151
TSH, mU/L	1.64±1.16	1.95 ±1.58	1.50 ±0.90	**0.015**
Estradiol (women) pg/mL,	62.62±79.06	61.41 ±82.6	63.2 ±77.95	0.531
Carotid Plaques	41 (20.5%)	12 (20.3%)	29 (20.4%)	1
IM complex	7.98±2.76	0.85 ±0.19	0.92 ±0.29	0.171

Abbreviations—THI: Tinnitus Handicap Inventory, VAS: Visual Analogue Scale; TTH: tension type headache, TSH: thyroid-stimulating hormone, HDL: high-density lipoprotein cholesterol LDL: low-density lipoprotein cholesterol, IM complex: intima-media complex.

The 25(OH)D level in the tinnitus group was significantly lower compared with the controls (p < 0.0001). In addition, significantly more patients in the tinnitus group were found to be deficient in vitamin D, compared with the controls (50.7% vs. 22.2%, respectively, p < 0.0001). Notably, only 8.5% of tinnitus patients had an optimal 25(OH)D level. In patients with severe tinnitus loudness (VAS > 5), the 25(OH)D serum level was significantly lower than in patients with mild tinnitus loudness (VAS < 5) (p < 0.0004). In patients with mild and moderate tinnitus severity (THI<57) the 25(OH)D serum level was significantly higher than in patients with severe tinnitus (THI>57) (p<0,0001). Similarly, patients with intermittent tinnitus had a trend towards a decreased 25(OH)D serum level compared with patients with continuous tinnitus (p < 0.0751). There were no differences in the 25(OH)D level between man and woman, younger and older, unilateral and bilateral localization of tinnitus (Table 3). In addition, the 25(OH)D level was not related to the coexistence of vertigo, headache, hearing loss, and mood disorders in tinnitus patients.

**Table 3:** Differences in the 25(OH)D level in tinnitus patients depending on gender, age, tinnitus parameters, and the presence of coexisting disorders.

Parameters	25(OH)D Level (ng/ml)			
	Mean	Std	Median	p-value
Men	19.07	6.49	18.60	0.3042
Women	20.53	8.29	20.30	
Age ≤ 50	19.29	7.73	17.70	0.2894
Age > 50	20.42	7.32	20.35	
Tinnitus unilateral	20.27	7.72	20.20	0.4204
Tinnitus bilateral	19.41	7.32	19.60	
Tinnitus continuous	20.33	7.79	20.4	**0.0751**
Tinnitus intermittent	17.95	6.11	17.15	
Tinnitus in ear	20.13	7.44	19.95	0.2820
Tinnitus in head	18.54	7.93	17.30	
Tinnitus ≤ 6 mc	20.86	6.81	20.20	0.4911
Tinnitus >6 mc	19.76	7.60	19.90	
VAS ≤ 5	22.17	7.51	22.80	**0.0004**
VAS > 5	18.12	7.09	17.50	
THI ≤ 57	22.22	7.10	22.60	**<0.0001**
THI > 57	15.24	6.09	14.30	
Loudness ≤ 40 dB	20.48	7.58	19.90	0.3031
Loudness > 40 dB	19.34	7.53	20.00	
Frequency ≤ 3000 Hz	19.90	7.62	20.05	0.8500
Frequency > 3000 Hz	20.11	7.51	19.90	
Hearing loss	17.92	7.39	18.30	0.1141
No hearing loss	20.25	7.52	20.20	
Headache	19.89	7.61	18.60	0.9519
No headache	19.82	7.47	20.40	
Vertigo	19.67	7.16	20.20	0.8852
No vertigo	20.10	8.03	18.50	
Hypertension	19.82	8.13	20.05	0.9724
No hypertension	19.87	7.28	19.90	

Tinnitus patients with a low level of 25(OH)D (≤15 ng/dl) were significantly younger, had a higher degree of tinnitus severity measured with THI and VAS scales, had higher triglyceride and TSH levels, and a lower HDL level, compared with individuals who had a higher 25(OH)D level (>15 ng/dl) (Table 2).

Having results presented in Tables 2 and 3, we decided to go a step further and prepare a multivariate logistic model to evaluate which set of independent variables could describe tinnitus best. We decided to use THI as the dependent variable describing tinnitus. The dependent variable in the regression model has two states: 0 –slight/mild/moderate (THI< = 57), 1 –severe/catastrophic (THI>57). Independent variables have been selected from the database and are: 25(OH)D level (< = 15, >15), gender (m/f), age (< = 50, >50), mood disorders: at least one from the group [sleep disorder, depression, anxiety] (yes/no), vertigo (yes/no), diabetes (yes/no), headache (yes/no), hearing loss (yes/no). From those factors (independent variables), optimal set of parameters has been selected to build a regression model. The process of selecting the optimal set of prognostic factors was performed using a backward selection procedure, starting with the model with all potential prognostic factors and eliminating irrelevant variables in subsequent steps. As a result of the analysis, three parameters were chosen: vitamin D level, age and mood disorders. P-values, Odds ratios (ORs) and corresponding 95% confidence intervals (CIs) for selected parameters are presented in Table 4.

**Table 4:** Multivariate logistic model evaluating independent variables which influence tinnitus.

Multivariate logistic regression model				
Parameter	OR	2.5% CI	97.5% CI	p-value
25(OH)D level	0.11	0.05	0.23	<0.0001
age	1.03	1.01	1.06	0.0179
Mood disorders	1.80	0.92	3.51	0.0846

Correlation analysis revealed that 25(OH)D level and THI as well as THI and VAS were strongly correlated (with correlation coefficients of -0.51 and 0.60, accordingly). There was also a significant but weak correlation between 25(OH)D and VAS (correlation coefficient: -0.22) (see S1 Fig).

Our study linked vitamin D deficiency with tinnitus, as only 8.5% of tinnitus patients had an optimal 25(OH)D level, while its level was significantly decreased in the tinnitus group when compared with healthy individuals. Moreover, the severity of tinnitus measured with THI and VAS scales correlated with the 25(OH)D level. Literature describes vitamin D intake association with reduced odds of hearing difficulties [27–29] and with tinnitus loudness variation [30]. Based on a literature review, our study is the first to demonstrate the high prevalence of vitamin D deficiency in patients with tinnitus and its effect on tinnitus parameters.

**Fig 1:**
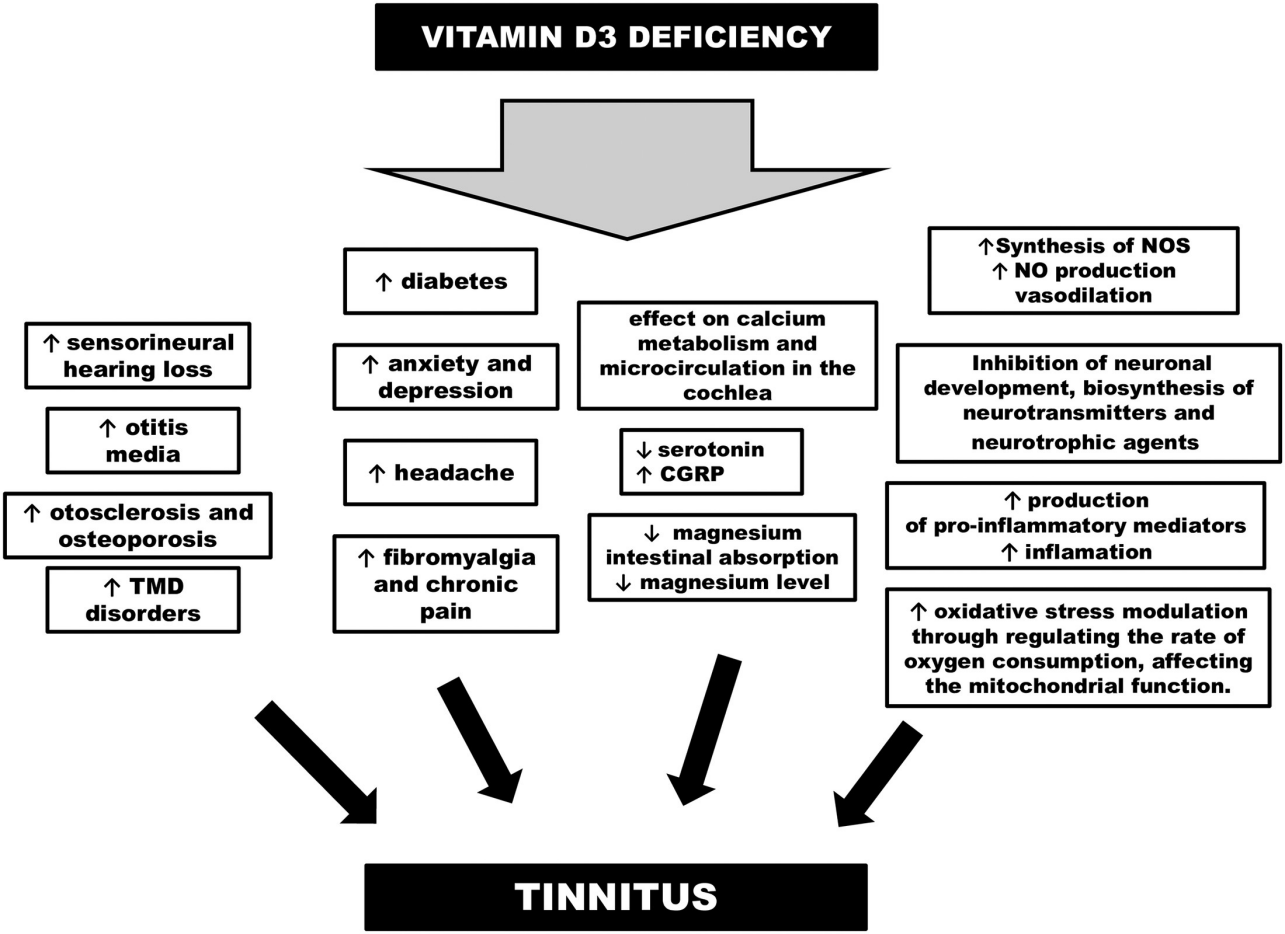
Proposed mechanisms by which vitamin D may influence tinnitus. Abbreviations: TMD temporo-mandibular disorders, CGRP—calcitonin gene-related peptide, NO—nitric oxide, NOS—nitric oxide synthases.

Considering our study results, the question arises about mechanism by which vitamin D affects tinnitus. Theoretically, there are a number of ways by which vitamin D may influence this disease (Fig 1, Table 5).

**Table 5:** Proposed mechanisms by which vitamin D may influence tinnitus.

	Author	Conclusion	Relation to tinnitus
1.	Ghazavi, H.; et al	Prevalence of vitamin D deficiency is higher in patients with SSNHL	SSNHL is tinnitus risk factor
2.	Shen, M.; et al Bousema, E. J, et al	Vitamin D deficiency may cause an erosive temporomandibular joint osteoarthritis	TMD is tinnitus risk factor
3.	Nowaczewska M, et al	A link between serum vitamin D levels and headaches/migraine	Headache/migraine is tinnitus risk factor
4.	Ellis at al	Association between vitamin D deficiency and fibromyalgia	Fibromyalgia is tinnitus risk factor
5.	Coomber, B, et al	Vitamin D reduces the production of nitric oxide (NO) by inhibiting the expression of NO synthase.	Endothelial dysfunction induces a dysfunction of microcirculation in the inner ear and may generate tinnitus
6.	Kim, H. B.; et al	Vitamin D deficiency may exacerbate inflammation and otitis media	Otitis media is tinnitus risk factor
7.	Uwitonze, A. M, et al	Magnesium is essential cofactor for vitamin D synthesis	Magnesium may have a beneficial influence on tinnitus

First of all, tinnitus may coexist with a great number of comorbidities and its prevalence may depend on many factors, such as sensorineural hearing loss, otitis media, otosclerosis, anxiety and depression, temporo-mandibular joint disorders (TMD), diabetes, dysthyroidism, pain, and headache [10, 31, 32]. Many of them are linked with vitamin D deficiency [13, 15, 18]. For example, the prevalence of vitamin D deficiency is higher in patients with sudden sensorineural hearing loss (SSNHL) than healthy individuals and SSNHL patients with deficient vitamin D had the highest percentage of no response to treatment [20]. Additionally, in humans, vitamin D deficiency has been associated with bilateral sensorineural hearing loss, possibly by interfering with the calcium metabolism and microcirculation in the cochlea [16, 33]. Vitamin D is known to have a direct effect on otoconia through controlling calcium concentration by regulating calcium absorption and uptake as well as ion channel expression [18]. Because sensorineural hearing loss is a risk factor for tinnitus development and the correlation between the generation of tinnitus and damaged hearing exist, 25(OH)D deficiency by inducing hearing loss may also contribute to tinnitus initiation or progression.

There is a bidirectional association between subjective tinnitus and TMD and evidence exists that vitamin D deficiency may cause an erosive temporomandibular joint osteoarthritis by stimulating the production of inflammatory cytokines [34, 35]. Contrary to these results, one study revealed that TMD patients had significantly higher values of vitamin D than controls [36].

As we already know, inflammatory mechanisms are involved not only in hearing loss but also in tinnitus pathogenesis [37]. Hence, the anti-inflammatory role of vitamin D may play an important part in tinnitus. There is an inverse link regarding the C-reactive protein (CRP, an inflammatory mediator) and vitamin D levels, and vitamin D supplementation can diminish inflammatory factors such as CRP [38, 39]. Also, some of vitamin D anti-inflammatory properties areconnected with reduction of the release of pro-inflammatory cytokines and inhibition T-cell responses [14]. Thus, altered cytokine production may be responsible for exacerbating the pathophysiological changes of otitis media in patients deficient in vitamin D [40]. Therefore, maintaining vitamin D status in the optimal range may be beneficial not only for proper management of otitis, but also for coexisting tinnitus. The risks of tinnitus were found to be significantly higher in patients with headache, especially migraine, as compared with those without headache [41, 42]. Our study revealed a high prevalence of headaches in the tinnitus group. On the other hand, many studies have shown a link between serum vitamin D levels and headaches, especially migraine, and some data indicate that vitamin D supplementation may be beneficial in some headache patients [15]. Regarding the non-coincidental relationship between tinnitus and headache, as well as the probable common pathophysiological mechanisms linked by both entities, one should expect that, similarly to headache sufferers, tinnitus patients would have a vitamin D deficiency and may benefit from vitamin D supplementation [43]. Fibromyalgia is another pain condition associated with tinnitus and vitamin D, as the incidence of tinnitus is high in fibromyalgia patients, and fibromyalgia treatment improves tinnitus [44]. Interestingly, a recent review showed an association between vitamin D deficiency and fibromyalgia, so both entities may be related to vitamin D [45]. Moreover, chronic pain is linked with vitamin D, and it is known that tinnitus and chronic pain share similar features regarding physiology, mechanisms, as well as assessment and management [46]. Osteoporosis is a common metabolic disorder that causes progressive changes in bone structure. Metabolic changes and possible degeneration of middle ear ossicles or the cochlear capsule may cause hearing loss in patients with osteoporosis. Kahveci et al. demonstrated a higher incidence of hearing loss and tinnitus complaints in patients with osteoporosis [47]. Besides, in a study evaluating the relationship between osteoporosis, balance, fall risk, and audiological parameters, tinnitus was more prevalent in the osteoporosis group compared with the controls [48].

Another mechanism by which vitamin D deficiency may influence tinnitus is connected with magnesium. Evidence exists that magnesium supplementation may lessen the severity of tinnitus and may have a beneficial influence on the perception of tinnitus-related handicap when scored with the THI [49]. Interestingly, magnesium plays a role as a main cofactor for vitamin D synthesis. Besides, activated vitamin D can enhance intestinal absorption of magnesium. On the other hand, supplementation of magnesium has beneficial effect on vitamin D activity [50, 51]. Therefore, diminished magnesium absorption due to vitamin D deficit may lead to tinnitus exacerbation. Vitamin D also diminish the production of nitric oxide (NO) by inhibiting the expression of NO synthase. NO is regulates neurotransmission and vasodilatation. As NO is involved in plastic neural changes associated with tinnitus and may contribute to tinnitus generation, vitamin D deficiency by increasing NO production and further endothelial dysfunction (which in turn induces a dysfunction of microcirculation in the inner ear) may generate tinnitus [52–54]. Moreover, there is evidence that serotonin is the most important hormone in tinnitus [55]. As vitamin D and its metabolites can influence many neurotransmitters, including serotonin, this may be another explanation for our study results [56]. In particular, vitamin D can regulate the synthesis of serotonin by tyrosine hydroxylase. Thus, in addition to its role in tinnitus pathogenesis, vitamin D deficiency may also cause depression, which often coexists with tinnitus. Another neuropeptide that plays a key role in synaptic plasticity and neurogenesis within inner ear structures is brain-derived nerve growth factor (BDNF) [3, 57]. Serum BDNF level was reported to be lower in tinnitus patients and may play a role in tinnitus etiology [58]. Vitamin D regulates the production of neurotrophic factors, including BDNF, so it can act as a neuroprotective agent in tinnitus patients [59]. A number of studies pay attention to the influence of oxidative stress on tinnitus: for example, the oxidative stress and antioxidant enzyme imbalance were more significant in tinnitus than in a control group, and tinnitus patients showed reduced effectiveness of the body’s natural antioxidant barrier compared to a control group [60, 61]. As vitamin D has a capacity to inhibit zinc-induced oxidative stress in the central nervous system, it may also act as an effective antioxidant to prevent tinnitus [62].

It is worth noting that, in our study, we found a significant, negative correlation between the level of vitamin D and the impact of tinnitus measured with THI and VAS scales, but not with tinnitus pitch and loudness. Indeed, many studies show that the VAS scale for tinnitus loudness does not correspond to psychoacoustic measures of tinnitus loudness, and there is no correlation between THI and pitch and loudness matching measurements [63, 64]. The reason for this discrepancy is that psychoacoustic measurements do not assess reactions to tinnitus, and self-reported tinnitus loudness is more a measure of tinnitus reactions than perception [65]. Why is vitamin D linked with tinnitus reactions and unrelated to tinnitus perception? First of all, tinnitus severity scores are closely related to psychological conditions of stress and depression in tinnitus patients [66]. On the other hand, vitamin D levels are significantly associated with the risk of anxiety symptoms and depression [67, 68]. Thus, it is possible that vitamin D deficiency by aggravating anxiety and depressive symptoms may influence reactions to tinnitus. As vitamin D supplementation was effective in ameliorating the severity of anxiety disorders, its effectiveness in tinnitus treatment cannot be excluded [69].

Surprisingly, we discovered that patients with intermittent tinnitus trended towards decreased 25(OH)D serum level compared with patients with continuous tinnitus. Usually, tinnitus is divided into an acute or a chronic persistent form. However, epidemiological studies show that intermittent tinnitus is the most common form [70]. Inner ear disorders are less frequent in patients with intermittent tinnitus as compared to those with chronic form [70]. Therefore, it is possible that, in individuals with intermittent tinnitus, vitamin D deficiency is one of the main contributing risk factors.

Another finding from our study is that tinnitus patients with vitamin D deficiency had higher triglyceride and TSH levels and a lower HDL level compared with individuals who had a higher 25(OH)D level. Indeed, there is evidence that vitamin D may have beneficial effects on serum lipid profiles, and its supplementation may diminish serum total cholesterol, LDL cholesterol, and triglyceride levels [71]. Some studies suggest that serum 25(OH)D is inversely correlated with levels of LDL cholesterol and triglycerides, and positively correlated with the level of HDL cholesterol [72, 73]. On the other hand, dyslipidemia is frequent in tinnitus patients, and tinnitus can be successfully dealt with by treating hyperlipidemia with lipid lowering agent atorvastatin [74, 75]. Thus, a link may exist between vitamin D, lipids, and tinnitus. Moreover, existing data show that tinnitus is also associated with thyroid disease history, and patients with hypothyroidism have shown a higher incidence of tinnitus [75, 76]. Similarly, vitamin D is associated with thyroid diseases, for example 25(OH)D deficiency is significantly associated with the degree and severity of hypothyroidism [77]. As in our study, we found that the THS level was higher in tinnitus patients with vitamin D deficiency, there may also be links between tinnitus, 25(OH)D, and thyroid function. On the other hand, high triglyceride and TSH levels may be related to sun exposure and/or physical activities. As 25(OH)D deficiency may also be influenced by both sun exposure and physical activity, we can’t exclude that it could have impact on our results.

Our study also revealed that tinnitus patients with a low level of 25(OH)D were significantly younger compared with individuals who had a higher 25(OH)D level. Indeed, it is reported that younger adults have a higher prevalence of vitamin D deficiency compared to older participants [78]. On the other hand, young people less frequently have arteriosclerosis, hypertension, diabetes, hearing loss and other factors which may influence tinnitus. Thus, in younger people vitamin D deficiency may be the main tinnitus risk factor.

Despite of our efforts, this study may have limitations. We did not include information about patients/controls lifestyle, workplace, sun and noise exposure, physical activity and diet, all of which might influence the vitamin D status and tinnitus.

## 4. Conclusions

A large proportion of tinnitus patients suffer from vitamin D deficiency, and their level of 25(OH)D is decreased compared to controls. The level of vitamin D correlates with the tinnitus impact measured with THI and VAS scales. The mechanisms by which vitamin D may influence tinnitus need to be elucidated. Although there is a link between vitamin D and tinnitus, a larger study should be performed to assess if vitamin D supplementation may be beneficial to tinnitus patients and to determine the optimal dose of vitamin D to be used in these patients. Based on our study, there is enough evidence to recommend vitamin D assessment to all tinnitus patients.

## Supporting information

S1 Fig The correlation plots for vitamin D level and THI as well as THI and VAS (coefficient values and statistical scores). (DOCX)
