# Supplementary material for: The role of vitamin D in subjective tinnitus—A case-control study
Source: PLoS One. 2021 Aug 18;16(8):e0255482. doi: 10.1371/journal.pone.0255482 (PMC8372974; doi:10.1371/journal.pone.0255482)
Supplement: S1 Fig — (DOCX) [file pone.0255482.s001.docx]

**The correlation plots for Vitamin D level and THI as well as THI and VAS ( coefficient values and statistical scores)
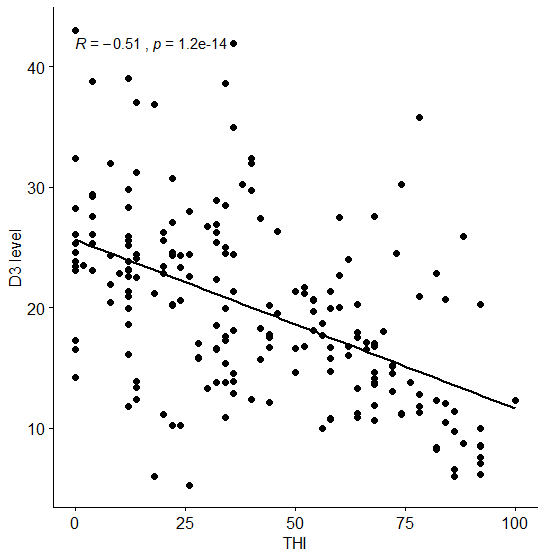

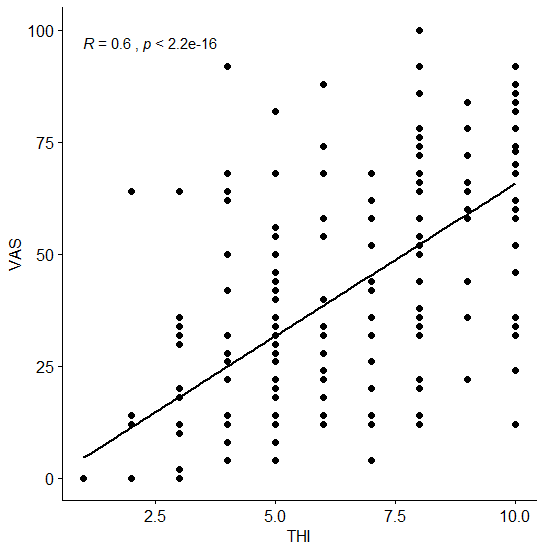
**
